# ACE2 Deficiency Enhances Angiotensin II-Mediated Aortic Profilin-1 Expression, Inflammation and Peroxynitrite Production

**DOI:** 10.1371/journal.pone.0038502

**Published:** 2012-06-05

**Authors:** Hai-Yan Jin, Bei Song, Gavin Y. Oudit, Sandra T. Davidge, Hui-Min Yu, Yan-Yan Jiang, Ping-Jin Gao, Ding-Liang Zhu, Guang Ning, Zamaneh Kassiri, Josef M. Penninger, Jiu-Chang Zhong

**Affiliations:** 1 State Key Laboratory of Medical Genomics, Ruijin Hospital, Shanghai Jiao Tong University School of Medicine, Shanghai, China; 2 Shanghai Key Laboratory of Vascular Biology, Shanghai Institute of Hypertension, Shanghai, China; 3 Divsion of Cardiology, Department of Medicine, Mazankowski Alberta Heart Institute, University of Alberta, Edmonton, Canada; 4 Departments of Obstetrics and Gynecology, Women and Children’s Health Research Institute, University of Alberta, Edmonton, Canada; 5 Department of Cardiology, Guangdong General Hospital and Guangdong Cardiovascular Institute, Guangzhou, China; 6 Institute of Molecular Biotechnology of the Austrian Academy of Sciences, Vienna, Austria; The Chinese University of Hong Kong, Hong Kong

## Abstract

Inflammation and oxidative stress play a crucial role in angiotensin (Ang) II-mediated vascular injury. Angiotensin-converting enzyme 2 (ACE2) has recently been identified as a specific Ang II-degrading enzyme but its role in vascular biology remains elusive. We hypothesized that loss of ACE2 would facilitate Ang II-mediated vascular inflammation and peroxynitrite production. 10-week wildtype (WT, Ace2^+/y^) and ACE2 knockout (ACE2KO, Ace2^−/y^) mice received with mini-osmotic pumps with Ang II (1.5 mg.kg^−1^.d^−1^) or saline for 2 weeks. Aortic ACE2 protein was obviously reduced in WT mice in response to Ang II related to increases in profilin-1 protein and plasma levels of Ang II and Ang-(1–7). Loss of ACE2 resulted in greater increases in Ang II-induced mRNA expressions of inflammatory cytokines monocyte chemoattractant protein-1 (MCP-1), interleukin (IL)-1β, and IL-6 without affecting tumor necrosis factor-α in aortas of ACE2KO mice. Furthermore, ACE2 deficiency led to greater increases in Ang II-mediated profilin-1 expression, NADPH oxidase activity, and superoxide and peroxynitrite production in the aortas of ACE2KO mice associated with enhanced phosphorylated levels of Akt, p70S6 kinase, extracellular signal-regulated kinases (ERK1/2) and endothelial nitric oxide synthase (eNOS). Interestingly, daily treatment with AT1 receptor blocker irbesartan (50 mg/kg) significantly prevented Ang II-mediated aortic profilin-1 expression, inflammation, and peroxynitrite production in WT mice with enhanced ACE2 levels and the suppression of the Akt-ERK-eNOS signaling pathways. Our findings reveal that ACE2 deficiency worsens Ang II-mediated aortic inflammation and peroxynitrite production associated with the augmentation of profilin-1 expression and the activation of the Akt-ERK-eNOS signaling, suggesting potential therapeutic approaches by enhancing ACE2 action for patients with vascular diseases.

## Introduction

Vascular inflammation and oxidative stress play a crucial role in the pathogenesis of vascular injury mediated by angiotensin (Ang) II, the major effector peptide of the renin-angiotensin system (RAS) [1]–[3]. Ang II has recently been shown to induce vascular injury by modulating release of inflammatory chemokines such as monocyte chemoattractant protein-1 (MCP-1), interleukin (IL)-1β, IL-6 [2], [3] and promoting the NADPH oxidase activation and superoxide (O_2_^–^) production [4], [5], contributing to endothelial nitric oxide (NO) synthase (eNOS) uncoupling and impaired NO bioavailability as well as vascular oxidative stress [1], [6], [7]. Within the vascular system, Ang II-mediated superoxide may drive a very fast radical-radical reaction by reacting with NO to yield the much more powerful oxidant peroxynitrite (ONOO^−^), which causes profound vascular injury via oxidative and nitrosative reactions [8]. In addition, Ang II is a well-known activator of profilin-1, extracellular signal-regulated kinases (ERK) and phosphatidylinositol 3-kinase (PI3K)/Akt signaling cascades, leading to increased formation of nitrotyrosine, an index of peroxynitrite damage to vascular tissues [1], [9], [10], [11], [12]. The actin-binding protein profilin-1 has been shown to directly activate Akt/ERK signaling pathways, which are important contributors of eNOS uncoupling and peroxynitrite generation in the vasculature [11], [13], [14]. However, little is known regarding the relationship among profilin-1, inflammation and peroxynitrite production in the vascular diseases.

Pharmacological inhibition of the Ang II signaling is a key aspect of the current approach to preventing vascular inflammation and oxidative stress-related vascular dysfunction [15]. Rencently, angiotensin-converting enzyme 2 (ACE2) has been identified as a pleiotropic monocarboxypeptidase responsible for the degradation of Ang II to the vasodilatory Ang-(1–7), which has been shown to counteract the pro-inflammatory and pro-oxidative effects of Ang II via its receptor Mas, thereby functioning as a negative regulator of the RAS [5], [16], [17], [18]. Intriguingly, the Ang II type 1 (AT1) receptor blockers may increase expression and activity of ACE2 [9], [19] with attenuation of cardiovascular oxidative damage [20] and diminish the profilin-1/ERK signaling [9], [10], [21]. In our previously studies, we demonstrated that ACE2 deficiency mice develop impaired cardiac functions, pathological remodeling with enhanced inflammatory cytokines, oxidative stress and ERK1/2 activation in heart [20], [22], [23] whereas ACE2 overexpression prevents Ang II-induced inflammation, oxidative stress and ERK1/2 activation in association with an attenuation of cardiovascular remodeling and dysfunction [9], [23], [24], suggesting a critical role ACE2 in regulation of inflammation, oxidative stress and cardiovascular function. However, the exact role and mechanism of ACE2 in vascular biology remain largely unknown. In this work, we assessed the hypothesis that loss of ACE2 would facilitate vascular inflammation and peroxynitrite production. We randomized ACE2 knockout (ACE2KO, Ace2^−/y^) and wild-type littermates (WT, Ace2^+/y^) mice to either Ang II or saline infusion as in the previous study [23], [24]. To investigate whether Ang II-induced pathological effects in the aortas of mice are mediated by AT1 receptor, we used irbesartan as a specific blocker of AT1 receptor.

## Materials and Methods

### Experimental Animals and Protocols

Mutant mice have been previously described [20], [22], [25]. 10-week male WT and ACE2KO mice received with mini-osmotic pumps (model 1002, Alza Corp, Palo Alto, CA) with Ang II (1.5 mg.kg^−1^. d^−1^) or saline for 2 weeks [24]. WT mice were treated with irbesartan (50 mg.kg^−1^.d^−1^; Bristol-Myers Squibb Co., Princeton, NJ) in their drinking water for 3 days before Ang II infusion and during the course of the study. Plasma levels of Ang II and Ang-(1–7) were measured by radio-immunoassay in the Hypertension and Vascular Disease Centre Core Laboratory at Wake Forest University School of Medicine as previously described [20], [25]. The thoracic aorta was carefully isolated from the mice anesthetized with a mixture (100 mg/kg ketamine plus 10 mg/kg xylaxine; ip) at the end of the experiment. All experiments were approved and performed in accordance with *the Guide for the Care and Use of Laboratory Animals* published by the US National Institutes of Health (NIH Publication No. 85-23, revised 1996), the Canadian Council on Animal Care and the Animal Research Ethics Committee at Shanghai Jiao Tong University School of Medicine.

### Dihydroethidium and Nitrotyrosine Fluorescence

Oxidative stress is generally identified by indirect markers of the vascular oxidant damage, such as superoxide and peroxynitrite production [6], [26]. The oxidative fluorescent dye dihydroethidium (DHE) and nitrotyrosine staining were used to evaluate superoxide (O_2_^–^) and peroxynitrite (ONOO^–^) levels in aorta tissues as previously described [23], [24], [26]. For aortic DHE staining, 20 µm fresh frozen tissue sections were washed with hanks balanced salt solution (HBSS) with magnesium and calcium and then incubated at 37°C for 30 min with DHE (10 µM) in HBSS. For nitrotyrosine staining, aortic tissue sections were immunostained using polyclonal nitrotyrosine antibodies (1∶100; Upstate Biotechnology Inc, Lake Placid, NY). Fluorescent images were observed with an Olympus Fluoview laser-scanning confocal microscope mounted on an Olympus microscope.

### Measurement of NADPH Oxidase Activity

The NADPH oxidase activities in aorta tissues of mice were quantified by lucigenin-enhanced chemiluminescence as previously described [20]. Briefly, the aorta homogenates were collected in 100 µl of phosphate buffer solution (PBS) mixture with protease inhibitor and phosphatase inhibitor and centrifuged at 1000 g for 10 min. The supernatants were then collected and added lucigenin (50 µM) and NADPH (1 mM) for NADPH oxidase activities assay with FB-12 luminometer in the absence or presence of diphenylene iodonium (10 µM), a selective inhibitor of NADPH oxidase. All data were calculated as the change in the rate of luminescence per minute per milligram of aorta tissue.

### TaqMan Real-time PCR Analysis

The mRNA expression levels were determined by TaqMan real-time reverse transcription PCR as previously described [5], [23], [27]. Total RNA was extracted from flash-frozen aorta tissues using TRIzol reagent, and cDNA was synthesized from 1 µg total RNA by using random hexamers. The primers and probes for mouse ACE2, MCP-1, IL-1β, IL-6, and tumor necrosis factor-α (TNF-α) are indicated in Table 1 based on previously published reports [5], [23], [27]. For each gene, a standard curve was generated using known concentrations of cDNA as a function of cycle threshold (CT). The mRNA expression of the reported genes was performed by TaqMan Real-time PCR using ABI 7900 Sequence Detection System and analyzed using the SDS2.2 software (Applied. Biosystems). All samples were run in triplicates. 18S rRNA was used as an endogenous control.

**Table 1:** Primers and probes sequences for TaqMan real-time PCR analysis*.

Genes	Primers/Probes	Sequences (Probe: 5′-FAM- -TAMRA-3′)
ACE2	Forward Primer	5′-GATACCTACCCTTCCTACATCAGC-3′
	Reverse Primer	5′-CTACCCCACATATCACCAAGCA-3′
	Probe	5′-CCACTGGATGCCTCCCTGCCC-3′
MCP-1	Forward Primer	5′-CCTGGATCGGAACCAAATGA-3′
	Reverse Primer	5′-CGGGTCAACTTCACATTCAAAG-3′
	Probe	5′-AACTGCATCTGCCCTAAGGTCTTCAGCA-3′
IL-1β	Forward Primer	5′-AACCTGCTGGTGTGTGACGTTC-3′
	Reverse Primer	5′-CAGCACGAGGCTTTTTTGTTGT-3′
	Probe	5′-TTAGACAGCTGCACTACAGGCTCCGAGATG-3′
IL-6	Forward Primer	5′-ACAACCACGGCCTTCCCTACTT-3′
	Reverse Primer	5′-CACGATTTCCCAGAGAACATGTG-3′
	Probe	5′-TTCACAGAGGATACCACTCCCAACAGACCT-3′
TNFα	Forward Primer	5′-ACAAGGCTGCCCCGACTAC-3′
	Reverse Primer	5′-TTTCTCCTGGTATGAGATAGCAAATC-3′
	Probe	5′-TGCTCCTCACCCACACCGTCAGC-3′

* Primer/probe mix for 18S were obtained from Applied Biosystems Inc. ACE2, angiotensin-converting enzyme 2; MCP-1, monocyte chemoattractant protein-1; IL1β, interleukin-1β; IL6, interleukin-6; TNFα, tumor necrosis factor-α.

### Western Blot Analysis

Western blot analyses were measured as previously described [12], [18]. Antibodies against ACE2 (90 kD), profilin-1 (15 kD), Akt (60 kD), ERK1/2 (44/42 kD), eNOS (140 kD), phospho-ERK1/2 (44/42 kD), phspho-p70S6 kinase (70 kD), phospho-Akt (Ser^473^; 60 kD), phospho-Akt (Thr^308^; 60 kD), phospho-eNOS (Ser^1177^;140 kD), nitrotyrosine (60 kD) and α-tubulin (55 kD) were obtained from R&D Systems (Minneapolis, MN), Santa Cruz Biotechnology (Santa Cruz, CA), and Cell Signaling Technology (Beverly, MA), respectively. Protein samples from thoracic aorta tissues were separated by 8%∼15% SDS-polyacrylamide gel electrophoresis and then transferred to PVDF membrane (Millipore). The membrane was blocked with 5% milk in Tris-Buffered Saline Tween-20 (TBST) for 2 h and then incubated overnight at 4°C with primary antibody. The membrane was washed thoroughly with TBST and then incubated with goat anti-rabbit IgG coupled to horseradish peroxidase (HRP) in TBST for l h at room temperature, then rinsed thoroughly with TBST as before. Aim proteins were detected by ECL using X-O-Mat X-ray film and analyzed by the ImageJ software (U.S. National Institutes of Health, Bethesda, MD).

**Figure 1:**
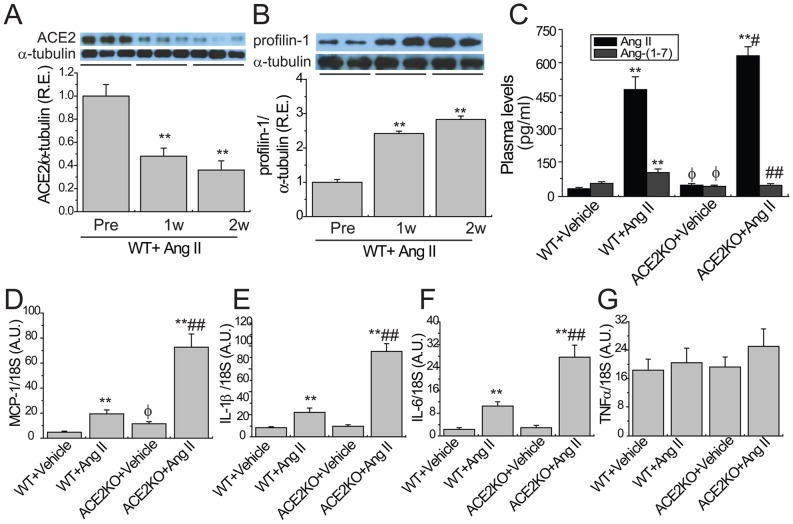
Ang II-mediated aortic expressions of ACE2, profilin-1, and inflammatory cytokines in mice. Representative Western blot analysis with quantification exhibited aortic expressions of ACE2 (A; 90 kD) and profilin-1 (B; 15 kD) at 0, 1 and 2 weeks after Ang II infusion in WT mice (n = 5). R.E. = relative expression; α-tubulin (55 Kd) was used as an endogenous control. ^**^ *P*<0.01 compared with pre-treatment group. Plasma Ang II and Ang-(1–7) levels (C) were measured in WT and ACE2KO mice in response to Ang II (n = 10). TaqMan real-time PCR analysis revealed the mRNA expressions of inflammatory cytokines including MCP-1 (D), IL1β (E), IL6 (F), and TNFα(G) in mice (n = 6−8). A.U. = arbitrary units; MCP-1, monocyte chemoattractant protein-1; IL1β, interleukin-1β; IL6, interleukin-6;TNFα, tumor necrosis factor-α. 18S rRNA was used as an endogenous control.^ **^ *P*<0.01 compared with corresponding vehicle-treated group; #, *P*<0.05 ##, *P*<0.01 compared with WT+Ang II group; φ, *P*<0.05 compared with WT+vehicle group.

### Statistical Analysis

All data are shown as mean±SEM. All statistical analyses were performed with SPSS 11.5 software either by Student’s t test or by ANOVA followed by the Student-Neuman-Keuls test for multiple-comparison testing as appropriate. A value of *P*<0.05 was considered statistically significant.

**Figure 2:**
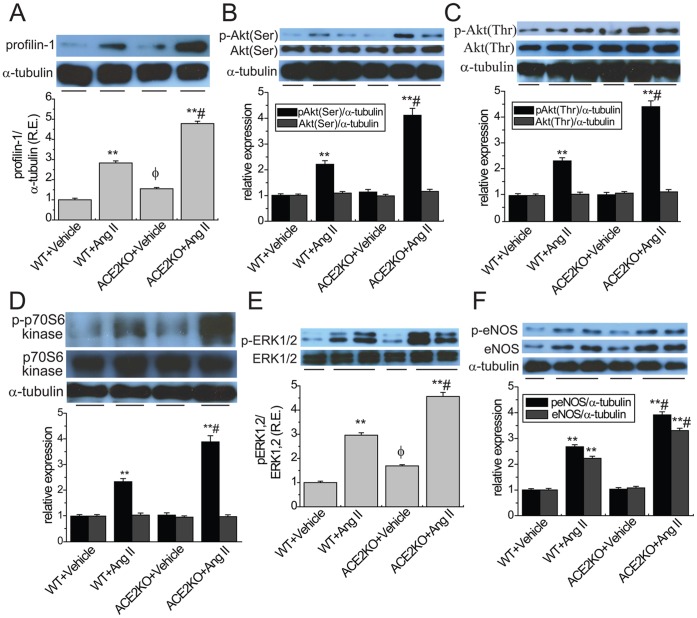
Ang II-mediated aortic profilin-1 expression and activation of signaling cascades in ACE2KO mice. Representative Western blot analysis with quantification exhibited aortic protein expression of profilin-1 (A; 15 kD) and phosphorylated levels of Akt at Ser^473^ and Thr^308^ (B & C; 60 kD), p70S6K (D; 70 kD), ERK1/2 (E; 44/42 kD) and eNOS (F; 140 kD) in WT and ACE2KO mice in response to Ang II (n = 5). R.E. = relative expression; ERK1/2, extracellular receptor-regulated kinases 1/2; eNOS, endothelial nitric oxide synthase. α-tubulin (55 kD) was used as an endogenous control. ^**^ *P*<0.01 compared with corresponding vehicle-treated group; #, *P*<0.05 compared with WT+Ang II group; φ, *P*<0.05 compared with WT+Vehicle group.

## Results

### ACE2 Deficiency Facilitates Ang II-mediated Aortic Inflammation

We firstly evaluated the effects of Ang II on aortic expressions of ACE2 and profilin-1. Western blotting revealed that aortic profilin-1 expression (Figure 1B) was obviously augmented at 1, and 2 weeks after Ang II infusion in WT mice compared with pre-treatment group (n = 6; *P*<0.01, respectively). This change was consistent with reduced protein of ACE2 (Figure 1A) and increased levels of plasma Ang II and Ang-(1–7) (Figure 1C) in WT mice (n = 6−10; *P*<0.01, respectively). Chronic Ang II infusion resulted in a marked increase in plasma Ang II levels (Figure 1C) without affecting plasma Ang-(1–7) levels in ACE2-deficient mice (n = 10; *P*<0.01, respectively). We next investigated Ang II-induced vascular inflammation in ACE2 deficiency status. TaqMan real-time PCR analysis revealed that lack of ACE2 resulted in greater increases in Ang II-induced mRNA expressions of inflammatory cytokines MCP-1 (Figure 1D), IL1β (Figure 1E) and IL6 (Figure 1F) in ACE2-dificient aortas (n = 6−8; *P*<0.01, respectively), without having a differential effect on the expression of TNF-α (Figure 1G).

ACE2 deficiency enhances Ang II-mediated aortic profilin-1 expression and the Akt/ERK/eNOS activation.

**Figure 3:**
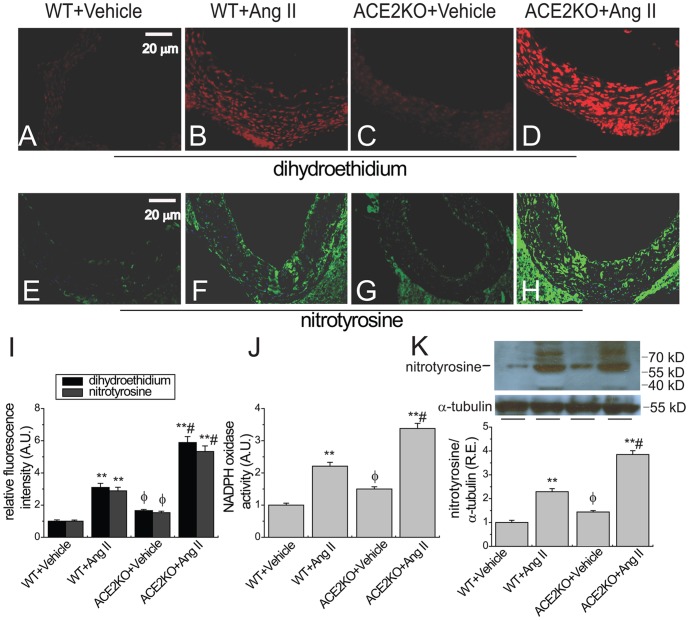
Ang II-mediated aortic superoxide and peroxynitrite generation in ACE2KO mice. Representative dihydroethidium (A–D) and nitrotyrosine (E–H) fluorescence images, relative fluorescence values (I) and NADPH oxidase activity (J) exhibited aortic superoxide generation and nitrotyrosine formation and NADPH oxidase activities in WT and ACE2KO mice in response to Ang II. Representative Western blot analysis showed protein level of nitrotyrosine (K; 60 kD) in aortas of WT and ACE2KO mice infused with Ang II. R.E. = relative expression; KO, knockout; A.U., arbitrary units; NADPH, nicotinamide adenine dinucleotide phosphate; α-tubulin (55 Kd) was used as an endogenous control. n = 5−6. ^**^ *P*<0.01 compared with corresponding vehicle-treated group; #, *P*<0.05 compared with WT+Ang II group; φ, *P*<0.05 compared with WT+Vehicle group.

As shown in Figure 2, increased expression of profilin-1 was exhibited in the aortas of ACE2KO mice associated with enhanced phospho-ERK1/2 level (n = 5; *P*<0.05, respectively) without affecting the expressions of phospho-Akt, Akt, phospho-p70S6K, phospho-eNOS and eNOS. Ang II infusion strikingly increased the aortic profilin-1 protein (Figure 2A) in both WT and ACE2 mutant mice (n = 5; *P*<0.01, respectively). Notably, in response to chronic stimulation by Ang II, there was a greater increase in profilin-1expression (Figure 2A) in the aortas of ACE2KO mice along with greater increases in the expression of phospho-Akt (Ser^473^; Figure 2B) and phospho-Akt (Thr^308^; Figure 2C) without affecting Akt (Figures 2B and 2C) as well as greater increases in phospho-p70S6 kinase (Figure 2D), phospho-ERK1/2 (Figure 2E), phospho-eNOS and eNOS (Figure 2F) (n = 5; *P*<0.05, respectively), indicating that loss of ACE2 resulted in greater increases in aortic profilin-1 expression and the activation of the Akt/ERK/eNOS signaling pathways.

**Figure 4:**
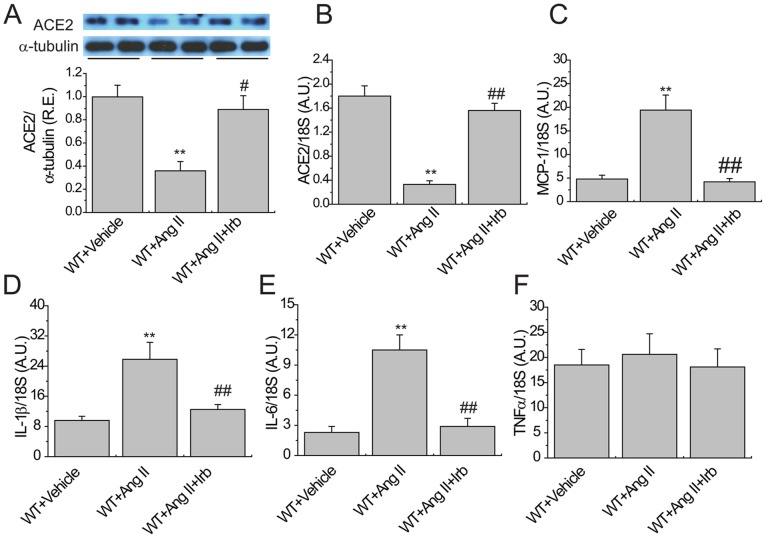
Aortic ACE2 expression and inflammation in Ang II-infused mice following irbesartan treatment. Representative Western blot analysis exhibited aortic protein levels of ACE2 (A) in WT mice (n = 5). α-tubulin (55 kD) was used as an endogenous control. TaqMan real-time PCR analysis showed the mRNA expression of ACE2 (B) and inflammatory cytokines MCP-1 (C), IL1β (D), IL6 (E), and TNF-α(F) in the aortas of WT mice (n = 6−8). A.U., arbitrary units; Irb, irbesartan; See Figure 1 for other abbreviations.^**^ *P*<0.01 compared with WT+vehicle group; #, *P*<0.05 ##, *P*<0.01 compared with WT+Ang II group.

### ACE2 Deficiency Worsens Ang II-mediated Aortic Peroxynitrite Production

Activation of NADPH oxidase is a central mediator of the pathological effects of Ang II, contributing to oxidative injury in the vasculature [8], [28], [29]. We found that chronic Ang II infusion significantly enhanced aortic NADPH oxidase activity (Figure 3J) and the production of superoxide (dihydroethidium fluorescence; Figures 3B, 3D & 3I) and peroxynitrite (nitrotyrosine staining; Figures 3F, 3H & 3I) in both WT and ACE2 mutant mice (n = 5−6; *P*<0.01, respectively). Furthermore, ACE2-dificient mice exhibited greater increases in aortic superoxide (Figures 3D & 3I) and peroxynitrite production (Figures 3H & 3I) associated with enhanced NADPH oxidase activity (lucigenin-enhanced chemiluminescence; Figure 3J) (n = 5−6; *P*<0.05, respectively). Interestingly, worsen aortic nitrotyrosine formation in ACE2-dificient mice was further confirmed by Western blot analyses showing a greater increase in protein level of nitrotyrosine (Figure 3L) in aortas in response to Ang II (n = 5−6; *P*<0.05, respectively), which indicated that loss of ACE2 facilitated Ang II-mediated peroxynitrite production in the vasculature.

**Figure 5:**
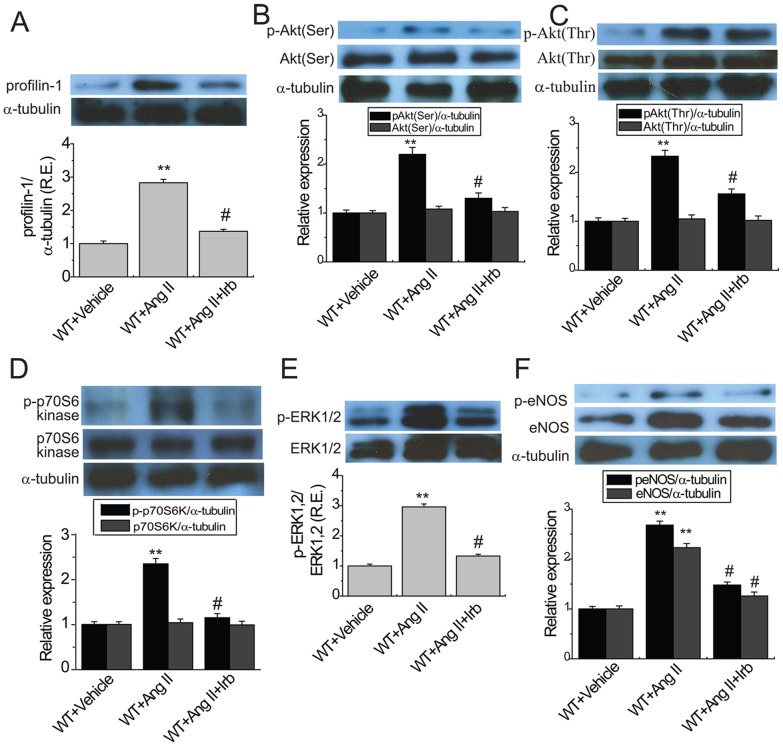
Aortic profilin-1 expression and signaling cascades in Ang II-infused mice following irbesartan treatment. Protein level of profilin-1 (A; 15 kD) and phosphorylated levels of Akt at Ser^473^ and Thr^308^ (B & C; 60 kD), p70S6 kinase (D; 70 kD), ERK1/2 (E; 44/42 kD) and eNOS (F; 140 kD) in aortas of WT mice using Western blot analyses. R.E. = relative expression; Irb, irbesartan; See Figure 2 for other abbreviations. α-tubulin (55 kD) was used as an endogenous control. n = 5. ^**^ *P*<0.01 compared with WT+vehicle group; #, *P*<0.05 compared with WT+Ang II group.

**Figure 6:**
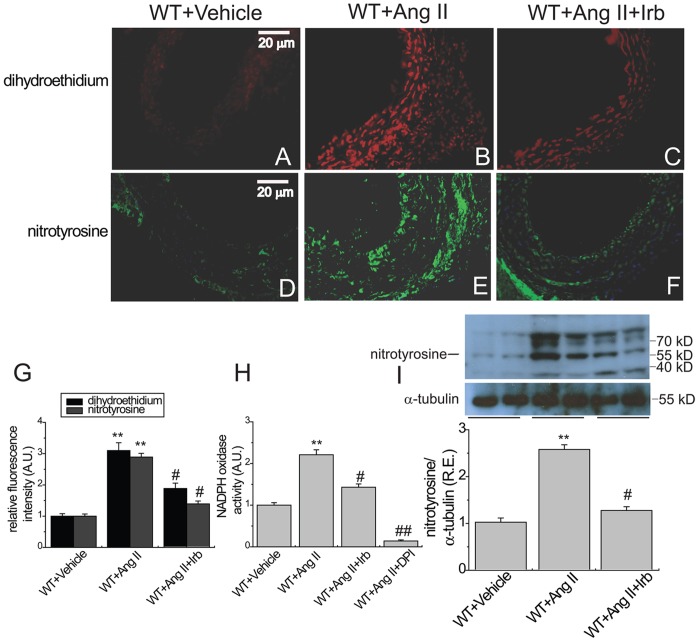
Aortic superoxide and peroxynitrite production in Ang II-infused mice following irbesartan treatment. Representative dihydroethidium (A–C) and nitrotyrosine (D–F) fluorescence images, relative fluorescence values (G) and lucigenin-enhanced chemiluminescence assay (H) exhibited the aortic superoxide and nitrotyrosine generation and NADPH oxidase activity in WT mice. Protein level of nitrotyrosine (I; 60 kD) was determined in aortas of WT mice by Western blot analysis. R.E. = relative expression; Irb, irbesartan; A.U., arbitrary units; NADPH, nicotinamide adenine dinucleotide phosphate; and DPI, diphenyleneiodonium. n = 5−6. ^**^ *P*<0.01 compared with WT+ vehicle group; #, *P*<0.05 compared with WT+Ang II group.

### AT1 Blockade Prevents Ang II-mediated Aortic Inflammation with the Altered ACE2 and Profilin-1 Signaling

Ang II is known to mediate its pathological effects in vascular system by means of the activation of AT1 receptors [20]. In this study, blockade of AT1 receptors by irbesartan treatment significantly upregulated the protein (Figures 4A) and mRNA (Figures 4B) expression of ACE2 while strikingly downregulated the mRNA expressions of inflammatory cytokines MCP-1 (Figure 4C), IL1β (Figure 4D) and IL6 (Figure 4E) without affecting TNF-α (Figure 4F) in aortas of WT mice (n = 5−8; *P*<0.05 or *P*<0.01, respectively). In addition, the Ang II-induced aortic profilin-1 expression (Figure 5A) was significantly prevented by AT1 receptor blockade (n = 5; *P*<0.05, respectively), associated with normalization of the Ang II-mediated increases in aortic expressions of phospho-Akt (Ser^473^; Figure 5B), phospho-Akt (Thr^308^; Figure 5C), phospho-p70S6 kinase (Figure 5D), phospho-ERK1/2 (Figure 5E), phospho-eNOS (Figure 5F) and eNOS (Figure 5F) in WT mice (n = 5; *P*<0.05, respectively). No significant differences were determined in the Akt expression among the groups (Figure 5B).

### AT1 Blockade Suppresses Ang II-mediated Aortic Peroxynitrite Production

Blockade of AT1 receptors by irbesartan treatment largely prevented Ang II-mediated increase in aortic nitrotyrosine staining (Figures 6F & 6G), superoxide production (Figures 6C & 6G) and protein level of nitrotyrosine (Figure 6I) in aorta of mice with suppression of NADPH oxidase activation (Figure 6H) (n = 5−6; *P*<0.05, respectively), providing that Ang II mediated activation of the AT1 receptor is a key pathophysiological event in the aortic superoxide and peroxynitrite production. Collectively, our data demonstrated that chronic Ang II infusion resulted in the AT1 receptor-mediated increases in superoxide and peroxynitrite production in the vasculature via the activation of the NADPH oxidase and the Akt-ERK-eNOS signaling pathways.

## Discussion

It is generally considered that peroxynitrite triggers cellular responses ranging from subtle modulations of cell signaling to overwhelming vascular oxidative injury and represents a crucial pathogenic mechanism of vascular diseases such as hypertension in experimental animal models and patients [1], [6], [8]. However, the molecular changes and signaling mechanisms still remain unclear in the peroxynitrite generation underlying Ang II-mediated vascular diseases. Within the vascular system, peroxynitrite is produced in all layers of the vascular wall by all vascular cell types, including endothelial, smooth muscle, and adventitial cells. They are formed by various enzyme systems, including uncoupled eNOS and NADPH oxidase, and others [1], [7], [8], [26], [29]. We have previously shown that loss of NADPH oxidase reduced Ang II-mediated superoxide production while enhancement of NADPH oxidase markedly augmented superoxide production in hearts of Ang II-infused ACE2 mutant mice [24], [28]. In the present study, we demonstrated that chronic Ang II infusion results in marked increases in aortic peroxynitrite production in both WT and ACE2 mutant mice with enhancement of superoxide production derived by NADPH oxidase activation. It has become clear that eNOS normally generates NO from L-arginine by utilizing NADPH [1], [29], [30]. Peroxynitrite may oxidize and disrupt the zinc thiolate center of eNOS by releasing zinc and oxidizing the thiols. This modification of eNOS (eNOS uncoupling) leads to diminishment of NO bioactivity and elevation of vascular superoxide production, which reacts with NO, further generating ONOO^−^ [8]. The present data suggest that the enhancement of NADPH-derived superoxide may act as a “kindling radical” to cause eNOS activation and peroxynitrite production in Ang II-infused mice. Ang II infusion increases, rather than decreases, the phosphorylation and protein levels of eNOS in both WT and ACE2-deficient mice. These findings are in line with previous observations in the rat model of oxidative stress mediated by Ang II [30] where eNOS expression was significantly upregulated in aorta tissues. More importantly, our current report exhibited that AT1 receptor blockade by irbesartan significantly attenuated Ang II-mediated aortic peroxynitrite production in WT mice, along with a marked reversal of Ang II-induced activation of NADPH oxidase and eNOS, confirming a critical contribution of Ang II/AT1 receptor-mediated activation of NADPH oxidase and eNOS to the peroxynitrite production.

The pivotal role of ACE2 as a negative regulator of Ang II-mediated signaling in the vascular system implies that ACE2 deficiency could facilitate the adverse effects of Ang II [15], [16], [17], [24], [28]. In the present study, aortic ACE2 protein is obviously reduced in WT mice in response to Ang II. This downregulation of ACE2 by Ang II in WT mice is related to marked increases in aortic profilin-1 protein. Moreover, absence of ACE2 leads to greater Ang II and lowered Ang-(1–7) levels in ACE2KO mice. In the Ang II-treated ACE2KO mice, loss of ACE2 triggers a greater increase in aortic profilin-1 expression linked with higher plasma Ang II levels. Ang II is critically involved in the activation of inflammatory cytokines, which may in turn trigger oxidative stress and cardiovascular dysfunction [2], [3], [27], [31], [32]. The key peptidase action of ACE2 is degradation of Ang II to Ang-(1–7), turning the balance within the RAS cascade from pro-inflammatory and pro-oxidative actions to anti-inflammatory and anti-oxidative actions [4], [12], [28], [31]. TaqMan real-time PCR analysis in this work revealed that lack of ACE2 results in greater increases in Ang II-induced mRNA expression of inflammatory cytokines MCP-1, IL1β and IL6 in ACE2-dificient aortas, without having a differential effect on TNF-α expression. The Ang II-induced aortic inflammation is significantly prevented by the AT1 receptor blocker irbesartan along with the suppression of peroxynitrite production and the enhancement of ACE2 expression. This is in agreement with other reports demonstrating that the enhanced ACE2 is associated with attenuation of Ang II-induced MCP-1 expression in cultured human monocyte cell line macrophages [33] and in aortas of the hypertensive rats [17]. Taken together, ACE2 deficiency facilitates Ang II-induced aortic inflammation and peroxynitrite production while enhanced ACE2 by AT1 receptor blockade is, at least in part, responsible for the attenuation of aortic inflammation and peroxynitrite generation.

There is growing evidence that profilin-1, a commonly recognized intracellular actin-binding protein, plays a prominent role in vascular pathology and vascular diseases [13], [14], [34]. Our current report for the first time demonstrated that ACE2 deficiency leads to greater increases in aortic profilin-1 expression in response to Ang II with the upregulation of eNOS in both phosphorylation and protein levels. It is not yet known whether this increase of profilin-1 is of pathophysiological significance in Ang II-mediated vascular diseases, but it has been revealed that profilin-1 overexpression leads to vascular inflammation [35] and vascular remodeling [14], [21]. Profilin-1 gene may represent a new therapeutic target in the treatment of vascular diseases such as hypertension [9], [10], [21]. Interestingly, profilin-1 has been shown to regulate eNOS phosphorylation and activity under atherogenic dietary and profilin-1 transgenic or mutant conditions [21], [34], [35]. In addition, profilin-1 interacts with signaling molecules including vasodilator-stimulated phosphoprotein (VASP), a validated marker for the activity of the eNOS/NO signaling pathway in vascular tissues [30]. Furthermore, profilin-1 triggers the activation of PI3K/Akt, which is an important regulator of vascular eNOS activation and peroxynitrite production through p70S6 kinase and ERK signaling pathways [2], [11], [13], [21]. In this work, Ang II-mediated increase in aortic profilin-1 expression is linked with augmented phosphorylation level of Akt at both regulatory sites, Ser^473^ and Thr^308^. In agreement with the pattern of Akt activation, phosphorylation levels of p70S6 kinase, ERK1/2, and eNOS are markedly augmented in WT and ACE2 mutant mice in response to Ang II, when compared with their corresponding control mice. Long-term blockade of AT1 receptors by irbesartan significantly augments the mRNA and protein expression of ACE2. More intriguingly, irbesartan provides protection against Ang II-mediated aortic peroxynitrite production via the suppression of profilin-1 expression and the Akt-ERK-eNOS signaling pathways in the present study. Consistent with our previous studies [9], these findings suggesting that Ang II-mediated upregulation of profilin-1 might, in part, contribute to the vascular peroxynitrite production.

Consequently, our findings demonstrate that ACE2 deficiency worsens Ang II-induced aortic inflammation and peroxynitrite production with the augmentation of profilin-1 expression and the activation of the Akt-ERK-eNOS signaling pathways, suggesting a critical role of ACE2 in the suppression of Ang II-mediated vascular injury and potential therapeutic approaches by enhancing ACE2 action for patients with vascular diseases. New roles for ACE2 may yet remain to be discovered and elucidated in the vascular biology. As highlighted in this work, further studies are required to better define the changes in vascular profilin-1 levels and the relative contribution and precise mechanism of profilin-1 in the inflammation and peroxynitrite production in the vasculature.
